# Quercetin stimulates trophoblast fusion via the mitochondrial function

**DOI:** 10.1038/s41598-023-50712-1

**Published:** 2024-01-02

**Authors:** Kanoko Yoshida, Kazuya Kusama, Go Shinohara, Shiho Sato, Mikihiro Yoshie, Kazuhiro Tamura

**Affiliations:** Department of Endocrine Pharmacology, Tokyo University of Pharmacy and Life Sciences, Tokyo, Japan

**Keywords:** Reproductive biology, Differentiation

## Abstract

The fusion of mononuclear trophoblasts into multinucleate syncytiotrophoblasts is the critical event in the process of syncytialization, and its dysregulation can lead to pregnancy complications, notably hypertensive disorders of pregnancy (HDP). Oxidative stress may disrupt trophoblast syncytialization in HDP. Specifically, placentas with HDP exhibit impaired mitochondria, giving rise to the generation of reactive oxygen species (ROS) and subsequent oxidative stress. Quercetin, a bioflavonoid known for its antioxidant and anti-aging properties, has the potential to mitigate oxidative stress during trophoblast syncytialization. However, the precise mechanism underlying the action of quercetin in these processes remains to be elucidated. To explore the impact of quercetin on syncytialization, mitochondrial function, and ROS generation, cyclic AMP-stimulated BeWo cells were treated with quercetin. The expression of markers associated with cell fusion, mitochondrial function, and oxidative stress was determined using qPCR and western blotting. Additionally, morphological syncytialization and mitophagy (mitochondrial degradation) were assessed by immunofluorescence analysis. Our results revealed that quercetin increased the expression of syncytialization markers and promoted cell fusion. Furthermore, this compound also upregulated markers associated with mitophagy and mitochondrial fusion, which are corroborated by visual evidence of mitophagy through the fluorescence microscope. Cell fusion naturally stimulated ROS generation, which was attenuated by quercetin. Quercetin downregulated the expression of NRF2 and HO-1 during syncytialization, while increasing the expression of sirtuin1/3/6, which are known to play essential roles in antioxidant responses. In conclusion, quercetin effectively regulates mitochondrial function through its antioxidant properties and the suppression of ROS generation, ultimately promoting trophoblast fusion, suggesting that the flavonoid has the potential to ameliorate pregnancy-related disorder stemming from placental dysplasia.

## Introduction

The placenta plays an essential role in maintaining pregnancy and fetal growth. The placenta is composed mainly of trophoblast cells which fuse to form multinucleated syncytiotrophoblast or differentiate into invasive extravillous trophoblasts (EVTs)^1^. The multinucleate syncytiotrophoblast contributes to the exchange of gases and nutrition, produces various bioactive factors, and protects the fetus from the maternal immune system^1,2^. Dysfunction of trophoblast syncytialization leads to the pathogenesis of pregnancy complications such as hypertensive disorders of pregnancy (HDP) and fetal growth restriction (FGR)^3^.

It has been recently reported that the pregnancy complications may be associated with the increase in oxidative stress and reduction of anti-oxidative capacity, accompanying the inhibition of trophoblast syncytialization^4,5^. Furthermore, the placenta with HDP exhibits the dysfunction of placental mitochondria such as a decrease in mitochondrial DNA expression and mitochondrial complex IV activity^6^. Mitochondria produces adenosine triphosphate (ATP) through oxidative phosphorylation, whereas induces various disorders by abnormal calcium transfer pathways and reactive oxygen species (ROS) produced during energy production, such as ATP^7,8^. Thus, mitochondrial disorders, mainly ROS generation, induce oxidative stress.

The Kelch-like ECH-associated protein 1 (Keap1)-nuclear factor-erythroid 2-related factor 2 (Nrf2) pathway plays an important defense system against ROS-induced oxidative stress. Nrf2 is a transcription factor that induces the expression of various defense genes and usually forms a complex with Keap1 dimers, and the transcriptional activity of Nrf2 is repressed by Keap1^9^. Heme oxygenase 1 (HO-1), also known as heat shock protein-32, is an inducible enzyme and is involved in the regulation of iron homeostasis and antioxidant activity^10^. These factors are important in understanding oxidative stress and have been reported to be involved in placentation. In response to ROS overproduction and oxidative stress, mitochondria continue to undergo morphological changes through repeated division and fusion, and hypofunction of mitochondria is degraded by mitophagy which is selective degradation through autophagy, which leads to various disorders accompanying the accumulation of abnormal mitochondria^11–13^. During the process of mitophagy, sequestosome1 (SQSTM1; known as p62) activates PTEN induced kinase 1(PINK1) / parkin RBR E3 ubiquitin protein ligase (PRKN) pathway, thus the decrease in SQSTM1 exhibits abnormal mitophagy^13^. In addition, PRKN localizes in mitochondria that lose their membrane potential, thereby promoting mitochondrial degradation^14^.

Quercetin (Que) is a bioflavonoid found in fruits and vegetables that has beneficial antioxidant, anti-inflammation, and anti-aging effects. Quercetin prevents cells from mitochondrial injury by regulating mitochondrial biogenesis, mitochondrial membrane potential, and ATP anabolism^15^. This compound may protect against aging-related diseases, through activating sirtuin1 (SIRT1), a member of an NAD^+^-dependent protein deacetylase^16^. Previous studies demonstrated that quercetin has removed senescent cells and accelerated differentiation of human endometrial stromal cells^17^. Furthermore, quercetin could have improved the dysfunction of trophoblast invasion by oxidative stress in EVTs^18^. However, the effect of quercetin such as the removal of oxidative stress and maintenance of mitochondria on trophoblast syncytialization remains unknown. In this study, we examined the effect of quercetin on the syncytialization and mitochondria function, especially mitophagy and ROS generation, in trophoblast BeWo cells.

## Results

### Quercetin potentiates the syncytialization of trophoblast

**Figure 1:**
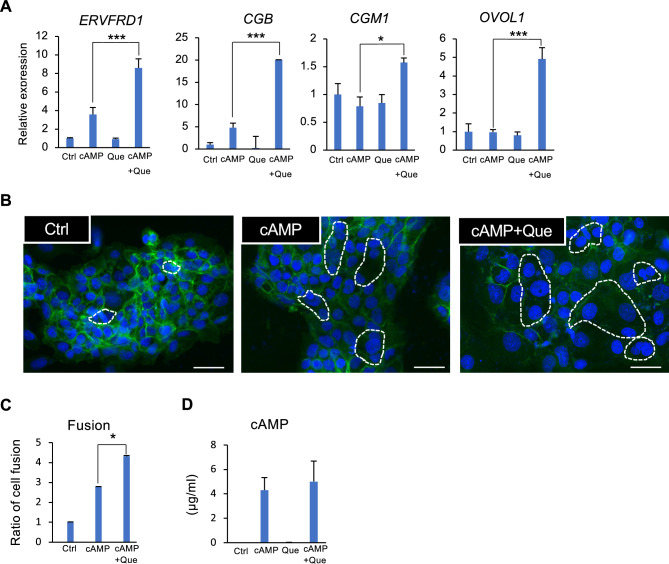
Quercetin (Que) potentiates the syncytialization of trophoblasts. BeWo cells treated with dibutyryl cyclic AMP (cAMP, 500 µM) and Que (5 µM) for 48 h. (**A**) Expression of syncytialization markers *ERVFRD-1*, *CGB*, *GCM1*, and *OVOL1* mRNA level was determined by qPCR. *GAPDH* was used as the reference gene. Values are represented as mean ± SEM of three independent experiments. *P < 0.05, ***P < 0.001. (**B**) Cells were immunostained with anti-E-cadherin antibody (green) and DAPI (blue) to visualize syncytialization. A representative picture from three independent experiments is shown and the syncytialized cells are marked with a stippled line. Scale bar = 100 µm. (**C**) Quantification of the number of syncytialized cells. The cell number in five areas selected randomly was counted in each experiment. The data are presented as ratios of the control and shown as mean ± SEM from three independent experiment, *P < 0.05. (**D**) Cyclic AMP level in the culture medium was determined by ELISA. Values are represented as mean ± SEM of three independent experiments.

To investigate the effect of quercetin on the syncytialization of trophoblasts, the trophoblast BeWo cells were treated with cyclic AMP analog (cAMP) and quercetin. The increase in intercellular cAMP produces chorionic gonadotropin subunit beta (CGB) and progesterone^19,20^, and upregulates fusogenic syncytin (ERVFRD-1) through the transcription factors, glial cell missing 1 (GCM1), and ovol like transcriptional repressor 1 (OVOL1) in trophoblast cells. Quercetin increased the expression of the syncytialization markers *ERVFRD-1, CGB, GCM1, OVOL1* and the number of fusogenic cells (Fig. 1A–C). Quercetin did not alter the intracellular cAMP levels (Fig. 1D).

### Quercetin promotes mitophagy and mitochondrial fusion markers in syncytiotrophoblasts

**Figure 2:**
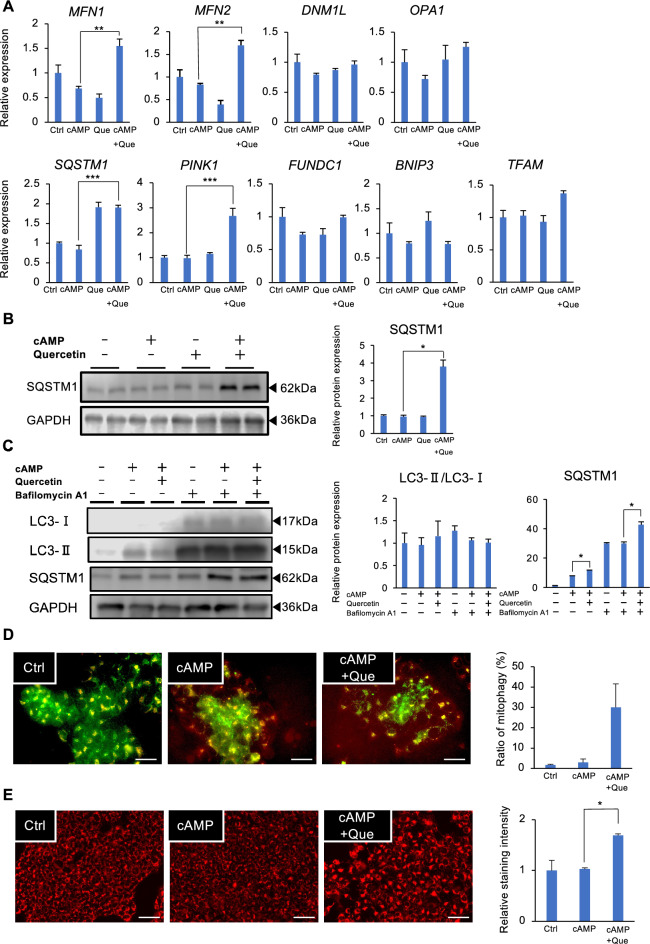
Quercetin (Que) promotes mitophagy and mitochondrial fusion and fission in trophoblasts. BeWo cells treated with dibutyryl cyclic AMP (cAMP, 500 µM) and Que (5 µM) for 48 h. (**A**) Expression of *MFN1/2*, *DNM1L*, *OPA1*, *SQSTM*1, *PINK1*, *FUNDC1*, *BNIP3*, and *TFAM* was determined by qPCR. *GAPDH* was used as the reference gene. Values are represented as mean ± SEM of three independent experiments. **P < 0.01, ***P < 0.001. (**B**) Immunoblotting showing the protein levels of SQSTM1 in lysates from cAMP- and Que-treated BeWo cells. GAPDH served as the loading control. Representative data from three independent experiments are shown. The graph shows SQSTM1 levels normalized to GAPDH levels from three independent experiments. *P < 0.05. Values represent mean ± SEM. (**C**) Proteins extracted from BeWo cells treated with cAMP, Que, and bafilomycin A1 (100 nM) for 48 h were subjected to immunoblotting. GAPDH served as the loading control. Representative data from three independent experiments are shown. The graph shows the ratios of LC3-II/LC3-I and SQSTM1/GAPDH from three independent experiments. *P < 0.05. Values represent mean ± SEM. (**D**) Assessment of mitophagy. Co-localization of the mitophagy dye with the lysosome dye was evaluated. A representative picture from three independent experiments is shown. Scale bar = 100 µm. The graph shows levels of mitophagy staining intensity from three independent experiments. Values represent mean ± SEM. (**E**) Assessment of mitochondrial membrane potential. A representative picture from three independent experiments is shown. Scale bar = 100 µm. The graph shows levels of membrane potential staining intensity from three independent experiments. Values represent mean ± SEM. *P < 0.05.

We investigated the molecular mechanism by which quercetin promoted syncytializaton in BeWo cells. Quercetin increased the expression of mitochondrial fusion markers *MFN1* and *MFN2* which is involved in the maintenance of mitochondrial morphology, but did not alter the expression of dynamin 1 Like (*DNM1L*), a mitochondrial fission marker^21^, and Optic Atrophy 1 (*OPA1*), a GTPase which is localized in the mitochondrial inner membrane^22^. Quercetin upregulated the expression of the mitophagy markers *SQSTM1* and *PINK1*, while did not alter other mitochondrial mitophagy markers *FUNDC1* and *BNIP3*, and mitochondrial biosynthesis proteins *TFAM* (Fig. 2A). Quercetin also increased SQSTM1 protein expression (Fig. 2B). To further evaluate mitophagy flux, bafilomycin A1 was used to block the mitophagic flux, resulting in the mitochondrial proteins, LC3-II and SQSTM1 being rescued by bafilomycin A1 (Fig. 2C). Effect of quercetin on the mitophagy of trophoblast was further examined using mitophagy (red) and lysosomal dyes (green). Mitophagy (as merged with red and green), the selective degradation of mitochondria by fusion with lysosomes, was observed in BeWo cells treated with quercetin (Fig. 2D). In addition, the co-treatment of quercetin and cAMP significantly increased mitochondrial membrane potential (Fig. 2E).

### Quercetin reduces mitochondrial ROS production and the expression of oxidative stress markers

**Figure 3:**
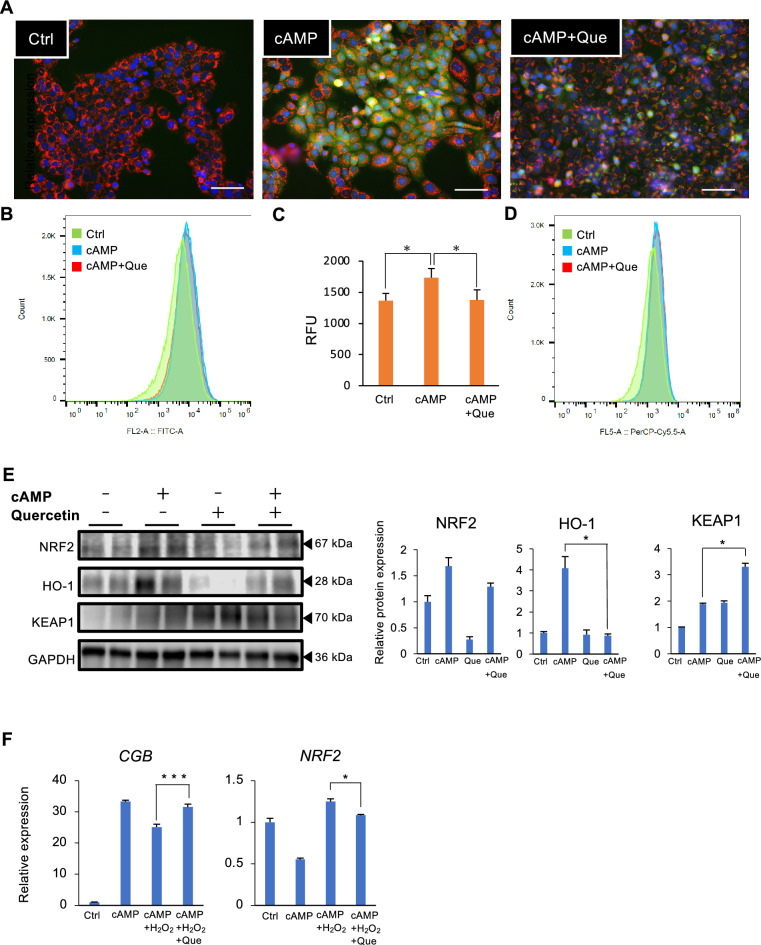
Quercetin (Que) reduces mitochondrial ROS production and the expression of oxidative stress markers in trophoblasts. BeWo cells treated with cAMP inducer forskolin (cAMP, 2.5 µM) and Que (5 µM) for 48 h. (**A**) Fluorescence micrographs of ROS. Cells were immunostained with anti-E-cadherin antibody (red), DAPI (blue) and DCFH-DA (green) to visualize ROS. A representative picture from three independent experiments is shown. Scale bar = 20 µm (**B**) Flowcytometry analysis of ROS. Representative data from three independent experiments are shown. (**C**) Intercellular level of ROS measured by DHFH-DA dye. *P < 0.05 vs. cAMP. Values are represented as mean ± SEM of three independent experiments. (**D**) Flowcytometry analysis showing the number of mitochondria. Representative data from three independent experiments are shown. (**E**) Immunoblotting of NRF2, HO-1, and KEAP1. GAPDH served as the loading control. Representative data from three independent experiments are shown. The graph shows NRF2, HO-1, and KEAP1 levels normalized to GAPDH levels from three independent experiments. *P < 0.05. Values represent mean ± SEM. (F) BeWo cells treated with dibutyryl cyclic AMP (cAMP, 500 µM) and Que (5 µM) for 48 h, and then H_2_O_2_ (400 µM) was added for 24 h. Expression of *CGB* and *NRF2* mRNA level was determined by qPCR. *GAPDH* was used as the reference gene. Values are represented as mean ± SEM of three independent experiments. *P < 0.05, ***P < 0.001.

To characterize the effect of quercetin on mitochondrial function, the production of ROS, the number of mitochondria, and oxidative stress were evaluated. Cellular fluorescence study showed that ROS was increased by cell fusion stimulation but decreased by quercetin treatment (Fig. 3A). Similar to Fig. 3A, data using flow cytometry (Fig. 3B) and microplate reader (Fig. 3C) displayed that cAMP-induced ROS level was inhibited by quercetin. In turn, the number of mitochondria was not changed regardless of cAMP and/or quercetin (Fig. 3D). Moreover, quercetin lowered the cAMP-stimulated NRF2 and HO-1 expression, whereas elevated KEAP1 (Fig. 3E). To further investigate whether quercetin affects these factors in the condition of increased ROS and/or mitochondrial damage, H_2_O_2_ as an oxidative stress inducer was treated. H_2_O_2_ decreased *CGB* and increased *NRF2* expression, however quercetin counteracted expression of *CGB* and *NRF2* (Fig. 3F).

### Quercetin upregulates SIRT1 expression in syncytiotrophoblasts

**Figure 4:**
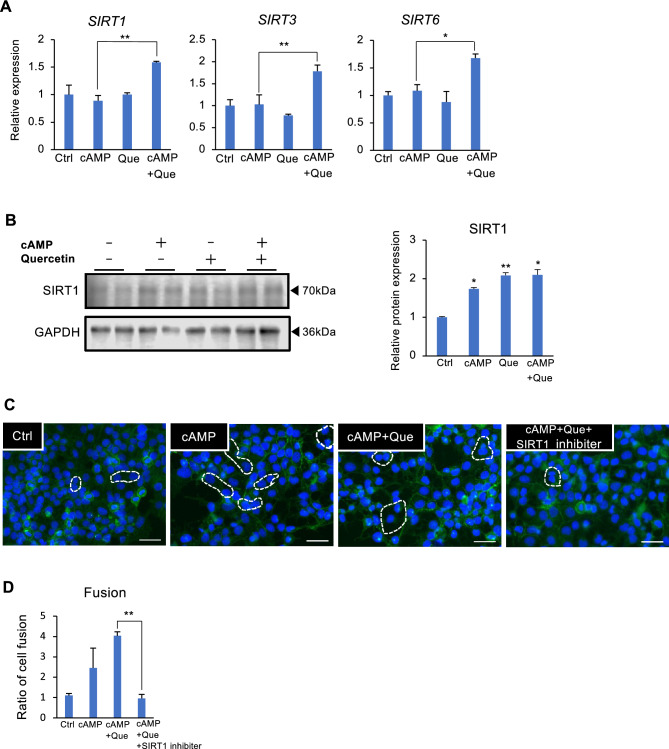
Quercetin regulates SIRT1 expression in trophoblasts. BeWo cells treated with dibutyryl cyclic AMP (cAMP, 500 µM) and Que (5 µM) for 48 h. (**A**) The expression of the *SIRT1*, *SIRT3*, and *SIRT6* was determined using qPCR analysis. *GAPDH* was used as the reference gene. Values are represented as mean ± SEM of three independent experiments. *P < 0.05, **P < 0.01. (**B**) Immunoblotting of SIRT1 in lysates from cAMP- and/or Que-treated cells. GAPDH served as the loading control. Representative data from three independent experiments are shown. The graph shows SIRT1 levels normalized to GAPDH levels from three independent experiments. Values represent mean ± SEM. *P < 0.05, **P < 0.01 *vs.* Ctrl. (C, D) BeWo cells treated with dibutyryl cyclic AMP (cAMP, 500 µM), Que (5 µM), and SIRT1 inhibitor (1 µM) for 48 h. Cells were immunostained with anti-E-cadherin antibody (green) and DAPI (blue) to visualize syncytialization and the syncytialized cells are marked with a stippled line (**C**). Scale bar = 100 µm. Quantification of the number of syncytialized cells (**D**). The cell number in five areas selected randomly was counted in each experiment. The data are presented as ratios of the control and shown as mean ± SEM from three independent experiment, **P < 0.01.

SIRT1 is expressed in human trophoblasts, exerts anti-oxidative stress and anti-inflammatory effects, but detail roles of SIRT1 in trophoblast remains unknown. We examined whether quercetin regulated the expression of SIRT1, SIRT3, or SIRT6 in trophoblast cells which are localized in the cytoplasm, mitochondria, or nucleus, respectively. The expression of SIRT1, SIRT3, and SIRT6 was not altered by cAMP alone, but was increased by co-treatment with quercetin (Fig. 4A). Similar to mRNA expression, SIRT1 protein expression was increased by quercetin (Fig. 4B). We further evaluated the effect of quercetin through SIRT1 on syncytialization using an SIRT1 inhibitor. SIRT1 inhibitor treatment significantly suppressed cell fusion by quercetin (Fig. 4C,D).

## Discussion

**Figure 5:**
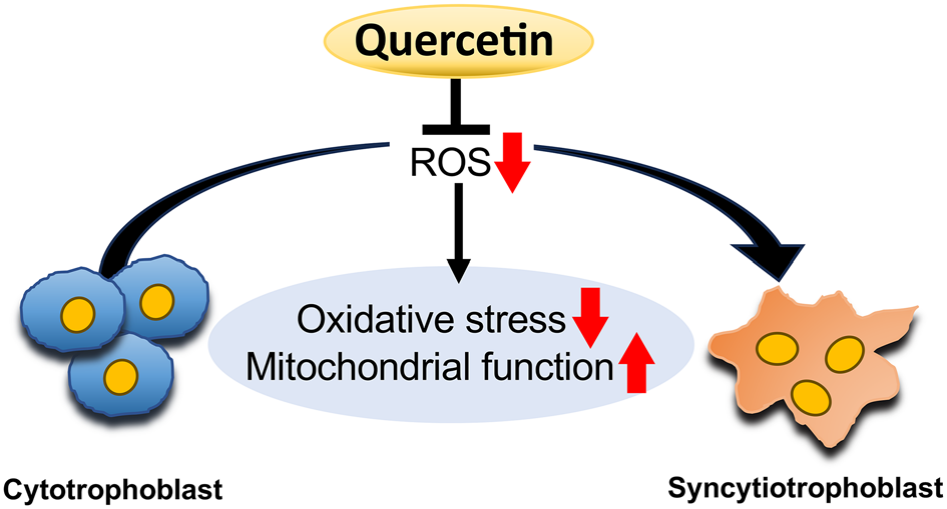
Schematic diagram illustrating the possible mechanism of quercetin action during trophoblast differentiation. Quercetin may inhibit oxidative stress caused by mitochondria-derived ROS. These effects could contribute to promotion of syncytialization.

This study showed that quercetin promotes syncytialization induced by cAMP. Furthermore, quercetin increased some mitochondrial function markers, mitophagy, and mitochondrial membrane potential, while decreased ROS production and oxidative stress markers during cell fusion. In addition, quercetin increased the expression of SIRT1, 3, and 6, which have been reported to inhibit ROS production and oxidative stress. Thus, quercetin may promote cell fusion via SIRTs expression by restoring mitochondrial dysfunction and oxidative stress associated with trophoblast syncytialization (Fig. 5).

In pregnant women, material metabolism and hormone levels dynamically change for the maintenance of pregnancy and fetal development, compared to non-pregnant women. During pregnancy, oxidative stress and endoplasmic reticulum (ER) stress have induced dysplasia of placenta^23^. The source of ROS during pregnancy is placental mitochondria^24^. In normal pregnancy, oxidative stress increases due to the generation of ROS that occurs alongside inflammatory response^8^. The present study first found that mitochondrial metabolism increased during syncytialization, by which the increase in ROS production caused oxidative stress in trophoblast cells. Furthermore, quercetin decreased ROS production and oxidative stress and increased the expression of SIRT1, 3, and 6 in trophoblasts. SIRTs inhibit ROS production and oxidative stress. Furthermore, SIRT3 has mitochondrial biosynthesis and antioxidant effects^25^, and SIRT6 is responsible for protecting and repairing damage from oxidative stress^26,27^. In addition, SIRT1 has been reported to be a protective molecule in trophoblasts with preeclampsia^28^.

On the other hand, ER stress may regulate the invasion of extravillous trophoblast cells and relate to the pathogenesis of HDP^29^. Previous study showed that the ER stress inducers tunicamycin and thapsigargin inhibited trophoblast fusion. However, treatment of quercetin under induction of ER stress did not change the expression of cell fusion markers (data not shown). These findings suggest that quercetin could remove oxidative stress which is harmful to pregnancy via partially SIRTs independent of ER stress.

In this study, the expression of the mitochondrial fusion markers MFN1 and MFN2 were increased by quercetin. However, some mitochondrial fission markers and the mitophagy marker BNIP3 were not changed. This indicates that quercetin acts on SQSTM1-induced PINK1 specific for mitophagy pathway and MFN1/MFN2 fusion markers to regulate morpho-functional maintenance of mitochondria. There are several mitophagy pathways besides the SQSTM1-induced PINK1 mitophagy pathway, of which the pathways via BNIP3L and FUNDC1 were not altered by quercetin, meaning that quercetin regulated mitochondrial functions mediated by the SQSTM1/PINK1 mitophagy pathway. It has been reported that the expression PINK1 is decreased in preterm FGR placentae and that the expression of MFN2 is decreased in the placentas of HDP patients^30,31^. These findings suggest that quercetin could have a therapeutic effect on a disorder of placentation. Further investigation of physiological function and molecular mechanisms of how quercetin affects mitochondrial morphology, degradation, and fusion on trophoblast syncytialization is required.

Administration of antioxidants during pregnancy may increase the risk for maternal health, and hence the development of safe active ingredients for pregnant women is required. HDP occurs in about 10% of pregnant women, and there is no fundamental cure, only delivery. Oxidative stress increases the risk of pregnancy complications and spontaneous abortion and increases the expression of oxidative stress markers in the tissue of HDP placentas^32^. It has been reported that impaired autophagy and increased mitochondrial damage were observed in preeclampsia placenta when compared with normal placenta^33^. Furthermore, our RNA-seq data of HDP placenta indicates that mitochondrial damage-related transcripts are changed (data not shown). In this study, quercetin decreased the expression of cell fusion- or H_2_O_2_-induced oxidative stress markers and induced mitophagy in trophoblast cells. Quercetin is contained in foods, and experiments with mice have confirmed that there is no teratogenicity in the fetus^34^. Therefore, quercetin may be an effective alternative in the prevention and treatment of pregnancy complications, such as HDP^35^.

In conclusion, quercetin effectively regulates mitochondrial function by exerting antioxidant effects, suppressing ROS generation via SIRTs during the cell fusion process, and resulting in the promotion trophoblast cell fusion. As a result, quercetin could improve normal placentation and potentially alleviate pregnancy complications associated with placental dysplasia.

## Materials and Methods

### Cell culture

The human choriocarcinoma BeWo cell line, purchased from the JCRB Cell Bank (Osaka, Japan), were grown in 1:1 Ham’s F12/Dulbecco’s modified Eagle’s medium (Fujifilm Wako Pure Chemical Corp., Osaka, Japan) supplemented with 10% FBS and 1% PSA (Fujifilm Wako Pure Chemical Corp.) at 37 °C in humidified air containing 5% CO_2_^36^. the cells were treated with dibutyryl cAMP (500 μM, Tokyo Chemical Industry, Tokyo, Japan) or forskolin (2.5 µM, Cayman chemical, Ann Arbor, MI USA) an adenylate cyclase activator, for 48 h to induce syncytialization. These cells were also treated for 48 h with Que (5 µM, Tokyo Chemical Industry).

### RNA extraction and quantitative RT-PCR

RNA was extracted using the RNeasy Mini Kit (Qiagen, Tokyo, Japan), according to manufacturer’s instructions. Reverse transcription of mRNA was performed using a ReverTra Ace qPCR RT Kit (Toyobo, Osaka, Japan), and the cDNA produced was subjected to qPCR amplification in a PowerUP SYBR Green Master Mix (Thermo Fisher Scientific, Waltham, MA, USA). The primers used are listed in Table S1. Calibration curves were used to determine the amplification of each target gene with respect to the expression of a reference gene, glyceraldehyde-3-phosphate dehydrogenase (GAPDH). The mean crossing threshold (Ct) values for each target were calculated using Sequence Detection System software v2.3 (Thermo Fisher Scientific)^37^.

### Cell fusion assay

BeWo cells were fixed with methanol and incubated with anti-E-cadherin antibody (1:200, #3195, CST) and AlexaFluor 594-conjugated goat anti-mouse antibody (Thermo Fisher Scientific) to distinguish cell surfaces. The nuclei were counterstained with 4′,6-diamino-2-phenylindole 2HCl (DAPI). The number of nuclei in syncytiotrophoblasts and total number of nuclei were counted in five randomly selected microscopic areas per sample, and the fusion index was calculated [(number of nuclei in syncytia/total number of nuclei) × 100] in three independent experiments^29^.

### ELISA for intracellular cAMP

BeWo cells (1.6 × 10^4^) were seeded in 96-well culture plates, and treated with dibutyryl cAMP (500 µM) and quercetin (5 µM) for 48 h. The concentration of cAMP in the culture medium was determined using the Cyclic AMP ELISA Kit (Abcam, Tokyo, Japan) according to the manufacturer’s instruction.

### Western blotting

Harvested cells were lysed in RIPA buffer (Thermo Fisher Scientific), and then equal amounts of lysate proteins were separated by SDS-PAGE and transferred onto polyvinylidene difluoride membranes (Bio-Rad Laboratories, Hercules, CA, USA) using a Trans-Blot Turbo (Bio-Rad). After blocking with Bullet Blocking One (Nacalai Tesque, Kyoto, Japan), the membranes were incubated with primary antibodies against SQSTM1 (1:5000, BC017222; ProteinTech, Chicago, IL, USA), NRF2 (1:2000, SAB1303359; Sigma-Aldrich, Tokyo, Japan), HO-1 (1:2000, ab13248; Abcam), KEAP (1:5000, BC002930; ProteinTech), SIRT1(1:5000, BC012499; ProteinTech) or GAPDH (1:5000, 5A12; Fujifilm Wako Pure Chemical Corp.). Immunoreactive bands were detected using enhanced chemiluminescence (Merck Millipore, Burlington, MA, USA) after incubation with horseradish peroxidase-labeled goat anti-rabbit or anti-mouse IgG (1:5000; Vector Laboratories, Burlingame, CA, USA). Signals were detected using a C-DiGit Blot Scanner (LI-COR), and the relative band density was quantified using Image Studio DiGit software (version 5.2)^38^.

### Mitophagy assay

BeWo cells (1.6 × 10^4^) treated with forskolin (2.5 µM), and quercetin (5 µM) were seeded into ibidi µ-slide 8well (Ibidi, Martinsried, Germany) coated with Matrigel (Corning, Corning, NY, USA) and cultured at 37 °C for 48 h. Mitophagy was detected using the Mitophagy detection kit (Dojindo Molecular Technologies, Kumamoto, Japan). The cells were treated with 100 nM Mtphagy Dye working solution at 37 °C for 30 min. After washing with the medium, cells were incubated with 1 µM Lyso Dye working solution for 30 min, and the levels of mitophagy were detected using fluorescence microscope. The fluorescence of colocalization was measured using the microcell count system (Keyence). Relative staining intensity was calculated [(fluorescence intensity of mitochondrial membrane potential/total staining area) × 100]. The data are presented as ratios of the control and shown as mean ± SEM from three independent experiments.

### Mitochondrial membrane potential assay

BeWo cells (4 × 10^4^) treated with forskolin (2.5 µM), and quercetin (5 µM) were seeded into 24-well coated with Matrigel (Corning) and cultured at 37 °C for 48 h. The mitochondrial membrane potential was evaluated using the MT-1 MitoMP Detection Kit (Dojin Molecular Technologies) following the manufacturer's protocol. Cells were incubated with MT-1 working solution for 30 min at 37 °C, and mitophagy were detected using BZX800 fluorescence microscope (Keyence). The fluorescence was measured using microcell count system (Keyence). Relative staining intensity was calculated (fluorescence intensity of mitochondrial membrane potential/total staining area). The data are presented as ratios of the control and shown as mean ± SEM from three independent experiments.

### The measurement of ROS level

BeWo cells (1.6 × 10^4^) treated with forskolin (2.5 µM), and quercetin (5 µM) were seeded into 96-well plates coated with Matrigel (Corning) and cultured at 37 °C for 48 h. The supernatant was removed, and a highly sensitive DCFH-DA dye working solution (Dojindo) was added and then incubated at 37 °C for 30 min. Changes in the levels of ROS were detected using fluorescence microscope, flow cytometry, and plate leader at Ex/Em: 490 nm/540 nm.

### Determination of the number of mitochondria

The number of mitochondria in BeWo cells using the MitoBright IM Red for immunostaining (Dojindo). BeWo cells (16 × 10^4^) treated with forskolin, and quercetin were seeded into 6-well plates cultured at 37 °C for 48 h. The alteration of the number of mitochondria was detected using flow cytometry.

### Statistical analysis

Data are expressed as mean ± SEM, and were compared using the Dunnett’s test. A *P*-value < 0.05 was considered to be statistically significant. Statistical testing was performed using the R software (ver.4.0.5; www.r-project.org).

### Supplementary Information

Supplementary Information.

## Data Availability

The datasets used and/or analyzed during the current study available from the corresponding author on reasonable request.
