# Supplementary material for: Quercetin stimulates trophoblast fusion via the mitochondrial function
Source: Sci Rep. 2024 Jan 2;14:287. doi: 10.1038/s41598-023-50712-1 (PMC10762005; doi:10.1038/s41598-023-50712-1)
Supplement: Supplementary file 1 — Supplementary Information. [file 41598_2023_50712_MOESM1_ESM.docx]

**SUPPLEMENTARY INFORMATION**

**Quercetin stimulates trophoblast fusion via the mitochondrial function.**

**Kanoko Yoshida, Kazuya Kusama*, Go Shinohara, Shiho Sato, Mikihiro Yoshie, Kazuhiro Tamura**

Department of Endocrine Pharmacology, Tokyo University of Pharmacy and Life Sciences

*Correspondence:

Kazuya Kusama Ph.D. kusamak@toyaku.ac.jp

**Table S1. Primers for real-time PCR analyses.**

| **Name**  **(Accession No.)** |  | **Sequence** |  | **Product length (bp)** |
| --- | --- | --- | --- | --- |
| *GAPDH* | F: 5'- | AGCCACATCGCTCAGACA | -3' | 66 |
| NM_002046.7 | R: 5'- | GCCCAATACGACCAAATCC | -3' |  |
| *ERVFRD-1* | F: 5'- | CCAAATTCCCTCCTCTCCTC | -3' | 115 |
| NM_207582.2 | R: 5'- | CGGGTGTTAGTTTGCTTGGT | -3' |  |
| *CGB* | F: 5'- | CCTGGCCTTGTCTACCTCTT | -3' | 108 |
| NM_000737.3 | R: 5'- | GGCTTTATACCTCGGGGTTG | -3' |  |
| *GCM1* | F: 5'- | GCAACACCAACAACCACAAC | -3' | 100 |
| NM_003643.3 | R: 5'- | GTAAATCTTGCGGCCTTCCT | -3' |  |
| *OVOL1* | F: 5'- | AGACATGGGCCACTTGACAG | -3' | 104 |
| NM_004561.3 | R: 5'- | AGGTGAACAGGTCTCCACTG | -3' |  |
| *MFN1* | F: 5'- | TTGGAGCGGAGACTTAGCAT | -3' | 71 |
| NM_001278465.2 | R: 5'- | TTGGAGCGGAGACTTAGCAT | -3' |  |
| *MFN2* | F: 5'- | ATTGCAGAGGCGGTTCGACTCA | -3' | 104 |
| NM_001127660.2 | R: 5'- | ATTGCAGAGGCGGTTCGACTCA | -3' |  |
| *DNM1L* | F: 5'- | AAACTTCGGAGCTATGCGGT | -3' | 66 |
| NM_001278465.2 | R: 5'- | AGGTTCGCCCAAAAGTCTCA | -3' |  |
| *OPA1* | F: 5'- | GCTCTGCATACATCTGAAGAACA | -3' | 52 |
| NM_130835.3 | R: 5'- | GCTCTGCATACATCTGAAGAACA | -3' |  |
| *SQSTM1* | F: 5'- | TGTGTAGCGTCTGCGAGGGAAA | -3' | 129 |
| NM_130835.3 | R: 5'- | AGTGTCCGTGTTTCACCTTCCG | -3' |  |
| *PINK1* | F: 5'- | GTGGACCATCTGGTTCAACAGG | -3' | 114 |
| NM_032409.3 | R: 5'- | GCAGCCAAAATCTGCGATCACC | -3' |  |
| *FUNDC1* | F: 5'- | AGACACCAGTGGTGGAATCGAG | -3' | 124 |
| NM_173794.4 | R: 5'- | TCTGGAACAGAAATCCTGCACAC | -3' |  |
| *BNIP3* | F: 5'- | TCAGCATGAGGAACACGAGCGT | -3' | 142 |
| NM_004052.4 | R: 5'- | GAGGTTGTCAGACGCCTTCCAA | -3' |  |
| *TFAM* | F: 5'- | AGCTCAGAACCCAGATGCAA | -3' | 238 |
| NM_003201.3 | R: 5'- | CCGCCCTATAAGCATCTTGA | -3' |  |
| *SIRT1* | F: 5'- | TAGACACGCTGGAACAGGTTGC | -3' | 117 |
| NM_001314049.2 | R: 5'- | CTCCTCGTACAGCTTCACAGTC | -3' |  |
| *SIRT3* | F: 5'- | CCCTGGAAACTACAAGCCCAAC | -3' | 162 |
| NM_001017524.3 | R: 5'- | CCCTGGAAACTACAAGCCCAAC | -3' |  |
| *SIRT6* | F: 5'- | TGGCAGTCTTCCAGTGTGGTGT | -3' | 142 |
| NM_001321064.2 | R: 5'- | CGCTCTCAAAGGTGGTGTCGAA | -3' |  |

F: Forward, R: Reverse.

**Figure S1. Full images of western blots shown in Figure 2.**

Red dotted lines indicate the cropping locations.

**
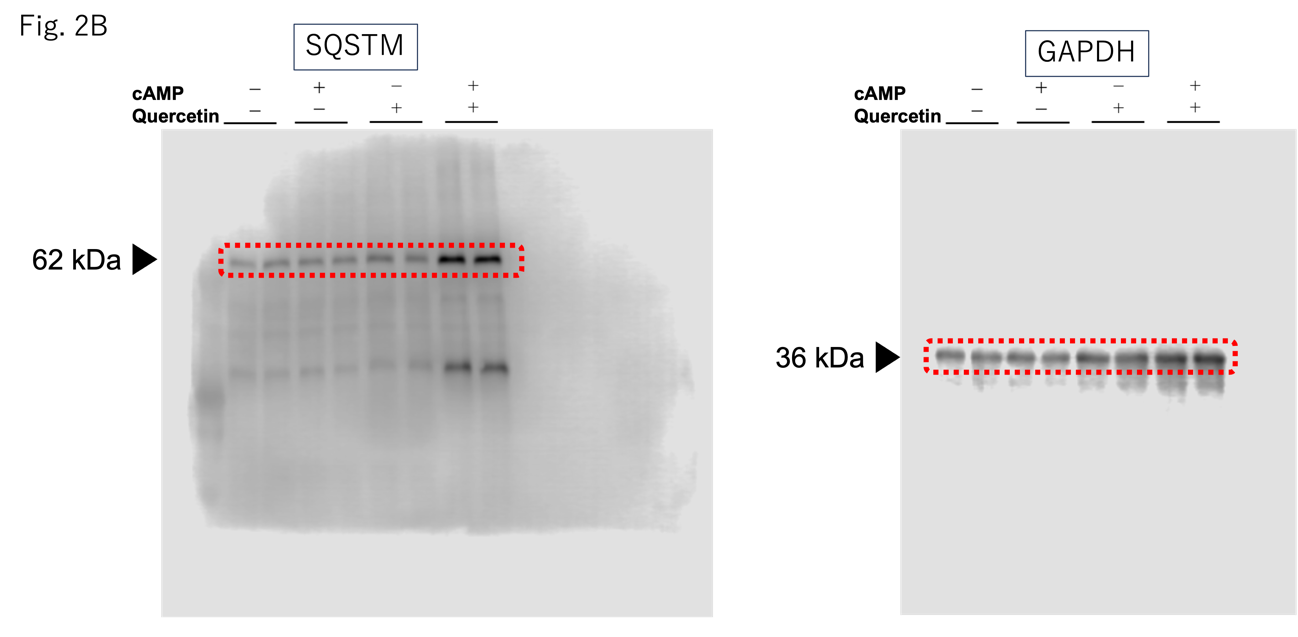
**


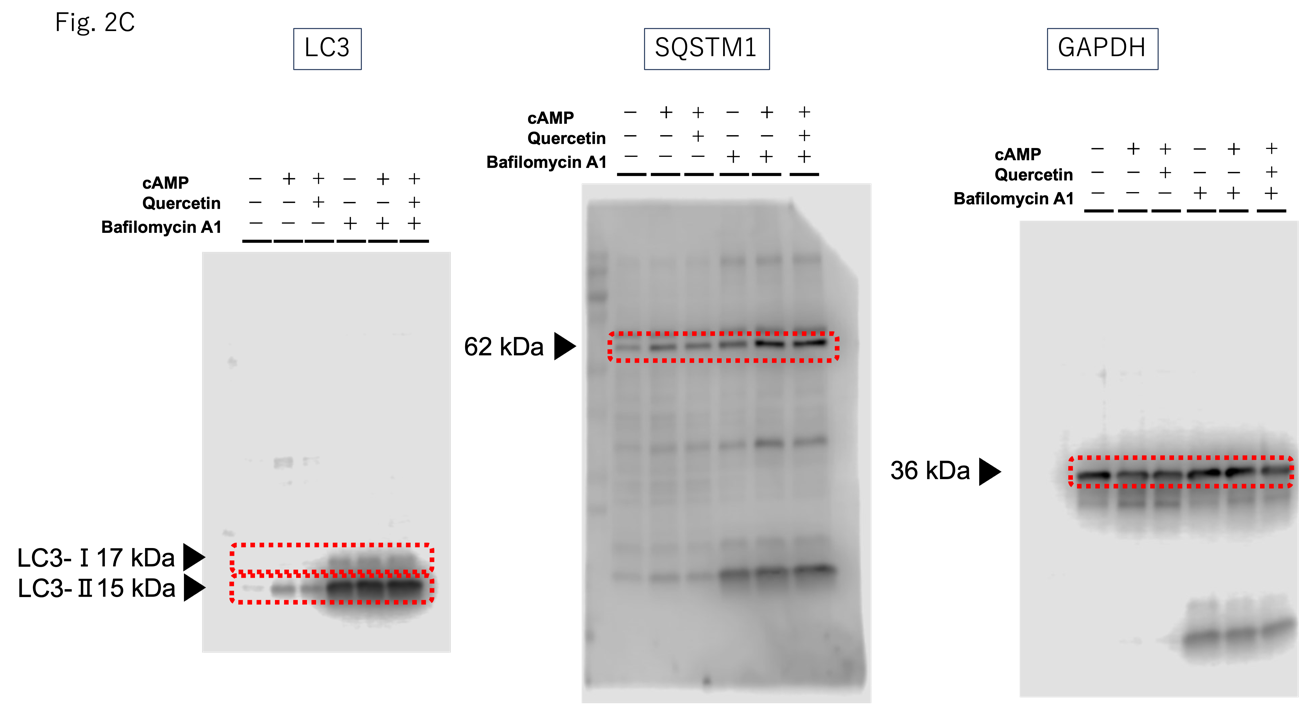


**Figure S2. Full images of western blots shown in Figure 3.**

**
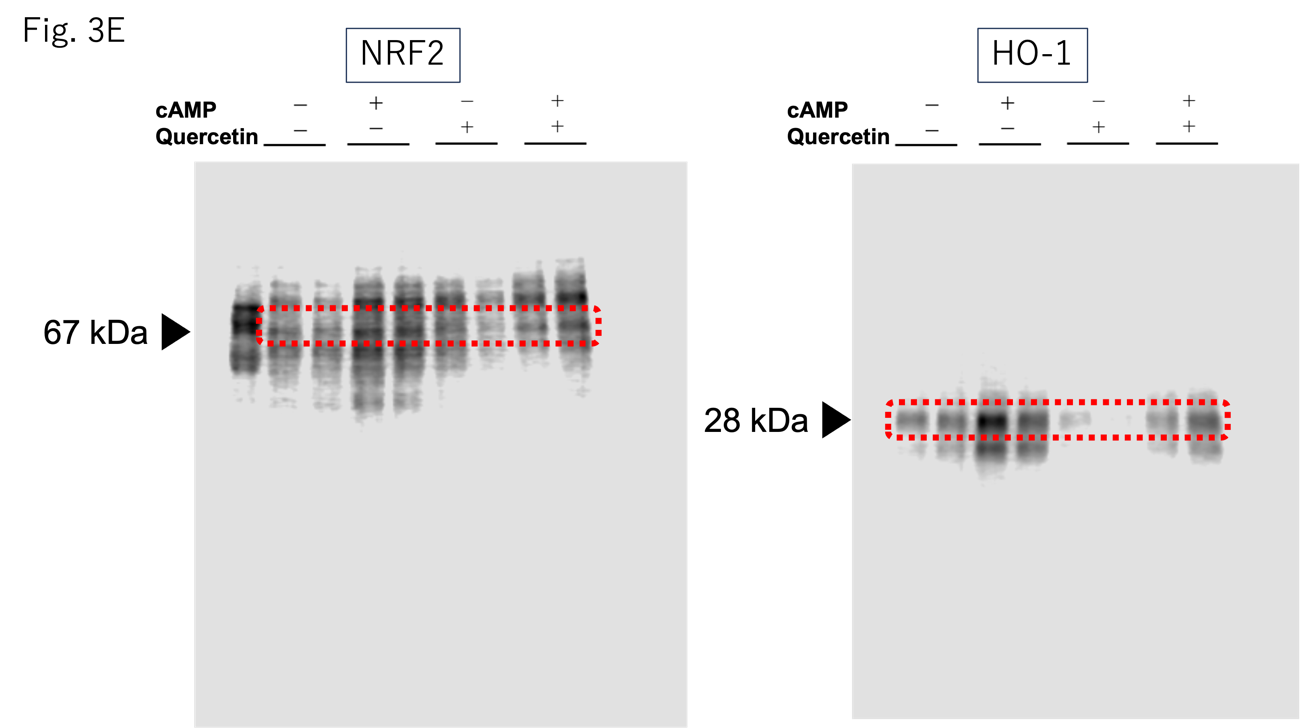
**

**
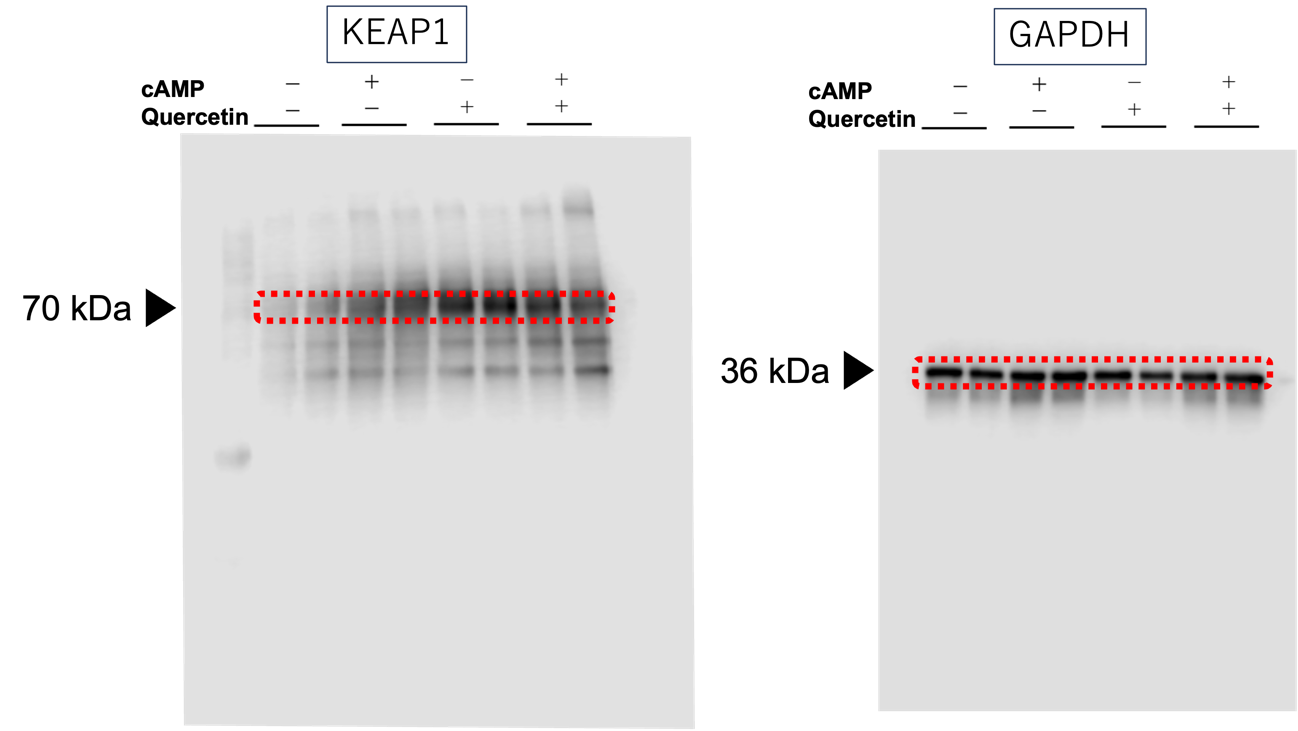
**

**Figure S3. Full images of western blots shown in Figure 4.**

**
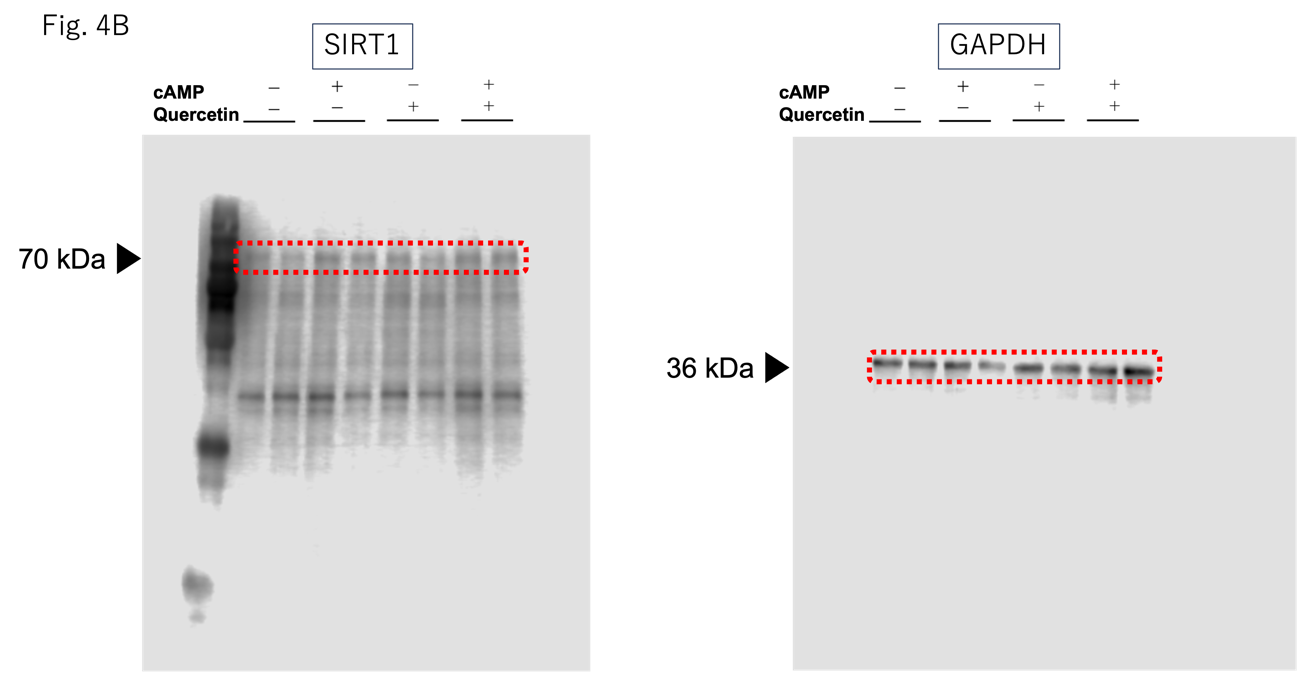
**
